# A structural and functional analogue of a Bowman–Birk-type protease inhibitor from *Odorrana schmackeri*

**DOI:** 10.1042/BSR20160593

**Published:** 2017-04-28

**Authors:** Yuxin Wu, Qilin Long, Ying Xu, Shaodong Guo, Tianbao Chen, Lei Wang, Mei Zhou, Yingqi Zhang, Chris Shaw, Brian Walker

**Affiliations:** 1 Natural Drug Discovery Group, School of Pharmacy, Queen’s University Belfast, Belfast BT12 6BA, Northern Ireland, U.K.; 2 Department of Nutrition and Food Science, College of Agriculture and Life Sciences, Texas A&M University, 123A Cater Mattil Hall, 2253 TAMU, College Station, TX 77843, U.S.A.; 3 Department of Emergency Medicine, The First Hospital of Hebei Medical University, Shijiazhuang 050031, China

**Keywords:** Bowman-Birk, Molecular cloning, Peptide, Skin secretion, Tryptase

## Abstract

Frog skin secretions contain complex peptidomes and peptidic protease inhibitors that are one of the biologically and structurally described groups of components. In the present study, by use of molecular ‘shotgun’ cloning and LC MS/MS fractionation sequencing, a novel Bowman–Birk-type heptadecapeptide (AALKGCWTKSIPPKPCF-amide), named *Odorrana schmackeri* Trypsin Inhibitor (OSTI), with a canonical Cys^6^–Cys^16^ disulfide bridge, was isolated and identified in piebald odorous frog (*O. schmackeri*) skin secretion. A synthetic replicate of OSTI-exhibited trypsin inhibitory activity with a *K*_i_ value of 0.3 ± 0.04 nM and also a tryptase inhibitory effect with a *K*_i_ of 2.5 ± 0.6 μM. This is the first time that this property has been reported for a peptide originating from amphibian sources. In addition, substituting lysine (K) with phenylalanine (F) at the presumed P1 position, completely abrogated the trypsin and tryptase inhibition, but produced a strong chymotrypsin inhibition with a *K*_i_ of 1.0 ± 0.1 μM. Thus, the specificity of this peptidic protease inhibitor could be optimized through modifying the amino acid residue at the presumed P1 position and this novel native OSTI, along with its analogue, [Phe^9^]-OSTI, have expanded the potential drug discovery and development pipeline directed towards alleviation of serine protease-mediated pathologies.

## Introduction

Inhibitors of serine proteinases are present in multiple forms in numerous tissues of animals and plants as well as in microorganisms, where they function by binding to their cognate enzymes in a substrate-like manner, forming stable complexes. Due to the fact that serine proteases perform some fundamental physiological roles such as peptide hormone release and blood coagulation, they are pathogenic factors in a variety of diseases which include pulmonary emphysema and cancers [1].

The Bowman–Birk-type inhibitor (BBI) family is a typical canonical serine protease inhibitor family, which was originally found in the seeds of leguminous (dicots) and gramineous (monocot) plants [2,3]. BBI proteins are usually small, cysteine (C) residue rich proteins, normally consisting of 60–90 amino acid residues and they share a high degree of structural homology. The rigid structures of BBI proteins are maintained by a series of highly conserved disulfide bridges. The majority of BBI proteins have a symmetrical ‘double-headed’ structure, each ‘head’ having two tricyclic domains and each domain has an independent canonical proteinase-binding site. One BBI molecule can form a 1:1:1 stoichiometric complex with two different proteinases [4].

The retention of trypsin or chymotrypsin inhibitory activity by fragmentation of the whole protein stimulated researchers’ interest in the minimal sequence requirements for bioactivity. Nishino et al. [5] synthesized a nine residue-based cyclic peptide that was recognized as the core antitrypsin loop: -C(P3)-T/A(P2)-X(P1)-S/A(P1′)-X(P2′)-P(P3′)-P/A(P4′)-Q(P5′)-C(P6′)-. Common with other canonical inhibitors, the residue at the P1 position is thought to be the main factor in determining the proteinases inhibitory activity and specificity [6]. Variants of BBI proteins generated by semisynthesis have shown that the phenylalanine (F) residue is the best choice for the P1 position for chymotrypsin inhibitory activity, while arginine (R) and lysine are preferred in the P1 position to inhibit trypsin [7]. Threonine (T) was proven to be the optimal residue at the P2 position when the peptide inhibits chymotrypsin based on the sequence –SCXFSIPPQCY-, because the side-chain of threonine has a dual role: through its side-chain –OH group, it mediates intraloop hydrogen bonding and through its –CH_3_ group, it mediates hydrophobic interaction between the enzyme and inhibitor [8].

Human tryptase has a trypsin-like specificity, it is the main protein in most human mast cells and constitutes approximately 20–25% of the total protein content of the cell. Tryptase is involved in inflammatory and allergic disorders, such as asthma, rhinitis, multiple sclerosis, psoriasis, interstitial cystitis and rheumatoid arthritis, and for this reason, tryptase inhibitors may have considerable therapeutic potential.

Until now, BBI peptides have been rarely reported from amphibian sources and our research group reported a novel C-terminally amidated BBI octadecapeptide from *Huia versabilis* in 2008 [9]. In 2014, another BBI peptide was reported from *Hylarana latouchii* [10]. All were found to have strong trypsin inhibition activities at nanomolar concentrations. Another study focused on the pLR/ranacyclin family member, named ORB, from the Oriental frog, *Odorrana grahami*, but this had a rather low trypsin inhibitory activity with a *K*_i_ value of 306 μM, suggesting either a less than optimal assay or a less than optimal peptide [11]. Here, we have isolated, identified and characterized a novel BBI peptide from the skin secretion of the piebald odorous frog, *Odorrana schmackeri* and named it *Odorrana schmackeri* Trypsin Inhibitor (OSTI). The peptide exhibited potent trypsin inhibitory activity and most importantly, showed strong tryptase inhibition effects, which is the first such activity reported for an amphibian skin peptide. In addition, we found that the analogue [Phe^9^]-OSTI exerted distinct chymotrypsin inhibition effects. Therefore, OSTI serves as a potential peptide template for the generation and design of candidate therapeutics for the treatment of gastrointestinal, dermatological and cardiovascular disorders.

## Materials and methods

### Specimen biodata and secretion harvesting

*O. schmackeri* (*n*=3, 5–7 cm snout-to-vent length, sex undetermined) were captured during expeditions in Fujian Province in the People’s Republic of China. All frogs were adults and secretion harvesting was performed in the field after which frogs were released. Skin secretion was obtained from the dorsal skin using gentle transdermal electrical stimulation [12]. The stimulated secretions were washed from the skin using deionized water and divided into either 0.2% v/v aqueous trifluoroacetic acid for subsequent peptide characterization or into cell lysis/mRNA stabilization buffer (Dynal) for subsequent cDNA library construction. Sampling of skin secretion was performed by Mei Zhou under UK Animal (Scientific Procedures) Act 1986, project license PPL 2694, issued by the Department of Health, Social Services and Public Safety, Northern Ireland. Procedures had been vetted by the IACUC of Queen's Universith Belfast, and approved on 1 March 2011.

### Reverse phase HPLC fractionation of skin secretion

The acidified skin secretion washings were clarified of microparticulates by centrifugation. The clear supernatant was subjected to reverse-phase HPLC fractionation using a Cecil Adept Binary HPLC system fitted with a Jupiter semi-preparative C-5 column (30 × 1 cm). This was eluted with a linear gradient formed from 0.05/99.95 (v/v) TFA/water to 0.05/19.95/80.0 (v/v/v) TFA/water/acetonitrile in 240 min at a flow rate of 1 ml/min. Fractions (1ml) were collected at minute intervals and the effluent absorbance was continuously monitored at λ_214_ nm. Samples (100 μl) were removed from each fraction in triplicate, lyophilized and stored at –20°C prior to biological activity analysis.

### Trypsin inhibition screening assay

A sample from each fraction was reconstituted using 22 μl of distilled/deionized H_2_O and each fraction was assayed in duplicate. All HPLC fractions were screened for trypsin inhibition. A volume of 180 μl of substrate working solution (Phe-Pro-Arg-NHMec, obtained from Sigma–Aldrich, Poole, Dorset, U.K.) (50 μM) was added to each row of 12 wells in a 96-well plate. The first two wells of each row contained substrate working solution only and acted as negative controls. A volume of 10 μl of each fraction sample was added to eight wells in each row. A volume of 10 μl of trypsin working solution (50 μM) was added to the remainder ten wells in each row to initiate the reactions. The plate was then placed into a fluorimeter to be analysed. The hydrolysis of the substrate by trypsin produced a fluorescent signal that was monitored at wavelengths of 460 nm for emission and 395 nm for excitation by a Fluostar Optima plate reader (BMG Labtech spectrofluorimeter).

### Identification and structural analysis of the predicted mature peptide OSTI

Molecular mass analysis of the components contained in the HPLC fraction exhibiting maximal trypsin inhibitory activity was achieved by use of a matrix-assisted laser desorption ionization time-of-flight (MALDI-TOF) mass spectrometer (Voyager DE, PerSeptive Biosystems, MA, U.S.A.). The major peptide within this fraction was subjected, without further purification, to MS/MS fragmentation sequencing using LCQ-Fleet mass spectrometer (Thermo Fisher, CA, U.S.A.). Finally, the primary structure of the novel trypsin inhibitory peptide was confirmed by molecular cloning that employed a specific primer designed from the MS/MS-derived amino acid sequence to interrogate an established *O. schmackeri* skin secretion derived cDNA library.

### Molecular cloning of OSTI cDNA from a cDNA library

Five milligrams of lyophilized skin secretion was dissolved in 1 ml of cell lysis/mRNA protection buffer obtained from Dynal Biotec, U.K. Polyadenylated mRNA was isolated from this by using magnetic oligo-dT Dynabeads as described by the manufacturer (Dynal Biotech, UK). The isolated mRNA was then subjected to 5′ and 3′ rapid amplification of cDNA ends (RACE) procedures to obtain full-length OSTI precursor nucleic acid sequence data using a SMART-RACE kit (Clontech, U.K.) as per manufacturer’s instructions. Briefly, the 3′-RACE reactions employed a nested universal primer (NUP) (supplied with the kit) and a degenerate sense primer (S: 5′-GCIGCIYTIAARGGITGYT-3′) that was complementary to the N-terminal amino acid sequence, A-A-L/I-K-G-C-W-, of the novel peptide, OSTI. The 3′-RACE reactions were purified and cloned using a pGEM-T vector system (Promega Corporation) and sequenced using an ABI 3100 automated sequencer. The sequence data obtained from the 3′-RACE product were used to design a specific antisense primer (AS: 5′-CCAAATTAGATGACTTCCAATTCAA-3′) to a defined conserved site within the 3′-non-translated region of the OSTI encoding transcript. 5′-RACE was carried out using these primers in conjunction with the NUP primer and resultant products were purified, cloned and sequenced.

### Solid-phase peptide synthesis of OSTI and [Phe^9^]-OSTI

Following confirmation of the primary structure of the novel cloned cDNA-encoded peptide, wild-type OSTI and its [Phe^9^]-OSTI analogue were successfully synthesized by standard solid-phase Fmoc chemistry using a Protein Technologies PS3™ automated peptide synthesizer. Following cleavage from the resin, deprotection and oxidative disulfide bond formation were performed.

The S–S oxidation was performed by adding 45 ml of diethyl ether into a 50-ml universal tube that contained the peptide and the universal tube was covered by a piece of pierced tinfoil and then exposed to the air for 3 days and shaken once every hour. The auto-oxidation process achieved by diethyl ether in the presence of oxygen mainly consisted of direct decomposition and radical isomerization [13].

### Reverse phase HPLC purification and primary structural confirmation of synthetic peptides

The synthetic peptides were analysed by both reverse phase HPLC (rpHPLC) and MALDI–TOF MS to establish degree of purity and authenticity of structure. The synthetic mixtures were purified and the primary structures of the major products (>95%) in each case, were subsequently confirmed by LC MS/MS.

### Trypsin, chymotrypsin and tryptase inhibition assays

Trypsin (10 μl from 0.1 μM stock solution in 1 mM HCl), was added to the wells of a microtitre plate containing substrate (Phe-Pro-Arg-NHMec) (50 μM) and synthetic peptide replicates (0.1–100 μM) in 10 mM phosphate buffer, pH 7.4, containing 2.7 mM KCl and 137 mM NaCl (final volume 210 μl).

Chymotrypsin (10 μl from 0.1 μM stock solution in 1 mM HCl) was added to the wells of a microtitre plate containing substrate (Succinyl–Ala–Ala–Pro–Phe–NHMec, obtained from Bachem, U.K.) (50 μM) and synthetic peptide replicates (0.1–100 μM) in 10 mM phosphate buffer, pH 7.4, containing 2.7 mM KCl and 137 mM NaCl (final volume 210 μl).

Tryptase (2.5 μl from 1 mg/ml stock solution, Calbiochem, U.K.), was added to the wells of a microtitre plate containing substrate (Boc-Phe-Ser-Arg-NHMec, obtained from Bachem, U.K.) (50 μM) and synthetic peptide replicates (0.5, 1, 2 and 4 mM) in tryptase assay buffer, pH 7.6, containing 0.05 M Tris, 0.15 M NaCl and 0.2% (w/v) PEG 6000 (final volume 210 μl).

Each determination was carried out in triplicate. The rate of hydrolysis of substrate was monitored continuously, at 37°C, by measuring the rate of increase in fluorescence due to production of 7-amino-4-methylcoumarin (NH_2_Mec) at 460 nm (excitation 360 nm) in a CytoFluor® multi-well plate reader Series 4000 spectrofluorimeter.

### Enzyme kinetics

For potent, slow, tight-binding inhibition, the Morison equation was used to determine the inhibition constant *K*_i(app)_ by fitting the kinetic data to the equation below [14]:

(1) $$\frac{V_{i}}{V_{0}}=1-\left\{\frac{{\left[\text{E}\right]}_{0}+ [\text{I}] +K_{i(app)}-\sqrt{{\left({\left[\text{E}\right]}_{0}+ [\text{I}] +K_{i(app)}\right)}^{2}-4\times {\left[\text{E}\right]}_{0}\times [\text{I}]}}{2\times {\left[\text{E}\right]}_{0}}\right\}$$

*V*_i_ and *V*_0_ are the velocities with and without peptides (inhibitors), [E]_0_ and [I] are total enzyme (trypsin) and peptides (inhibitors) concentrations respectively.

This kinetic treatment was used for the inhibition of trypsin by OSTI. Since the inhibition studies were performed in the presence of competing substrate, the true *K*_i_ value was calculated from *K*_i(app)_ using eqn (2). For trypsin, a fixed substrate concentration [S] of 50 μM was used, for these inhibition studies and in a separate experiment (performed using identical buffer and temperature conditions),a value of 73.29 μM was determined for the Michaelis constant *K*_M_, for substrate hydrolysis in the absence of OSTI.

(2) $$K_{i}=\frac{K_{i(\mathrm{app})}}{1+ [S]/K_{M}}$$

For modest binding inhibition, we first calculated the IC_50_ from a plot of % inhibition against inhibitor concentration [I]. The data points were fitted to the resulting curve, by non-linear regression analysis, using GraphPad Prism. The IC_50_ values were then converted into *K*_i_ values using the eqn (3), to account for the presence of competing substrate [15].

(3) $$K_{i}=\frac{{\text{IC}}_{50}}{\left\{1+\left(\left[\text{S}\right]/K_{M}\right)\right\}}$$

This kinetic treatment was applied to the inhibition of tryptase by OSTI and chymotrypsin by [Phe^9^]-OSTI. In inhibition studies with both these proteases, their respective substrates were used at a fixed concentration [S] of 50 μM. The Michaelis constant *K*_M_ for tryptase against Boc-Phe-Ser-Arg-NHMec was determined to be 227.2 μM, while *K*_M_ for chymotrypsin against Succinyl–Ala–Ala–Pro–Phe–NHMec was determined to be 38.4 μM. The determination of these *K*_M_ values was carried out using identical buffer and temperature conditions (37°C) as those employed in the inhibition studies with the two synthetic amphibian peptides.

### Determination of peptide secondary structures using CD analysis

CD measurements were performed with a JASCO J-815 CD spectrometer (Jasco, Essex, U.K.) at room temperature (20–25°C). Each peptide was dissolved in (a) water and (b) 50% (v/v) trifluoroethanol (TFE)-water to reach a concentration of 50 μM before being transferred and measured in a 0.1 cm high precision quartz cell (Hellma Analytics, Essex, U.K.). The wavelength used in CD spectrometer was from 190 to 240 nm with a scanning speed of 200 nm/min, bandwidth and data pitch are 1 and 0.5 nm respectively. CD data are expressed as the mean molar ellipticity [θ] in deg.cm^2^.dmol^−1^ in corresponding wavelength (nm), which is calculated using the measured ellipticity (θ, in medg) with the equation [θ] = θ/(10 × c × l), ‘c’ refers to the molar concentration of the sample (mol/l) and the term ‘l’ is the cuvette path length (cm). DichroWeb webserver (http://dichroweb.cryst.bbk.ac.uk/html/home.shtml) was used to estimate the α-helix and β-sheet content [16–18].

## Results

### Trypsin inhibitory activity of rpHPLC fractions of *O. schmackeri* skin secretion

**Figure 1:**
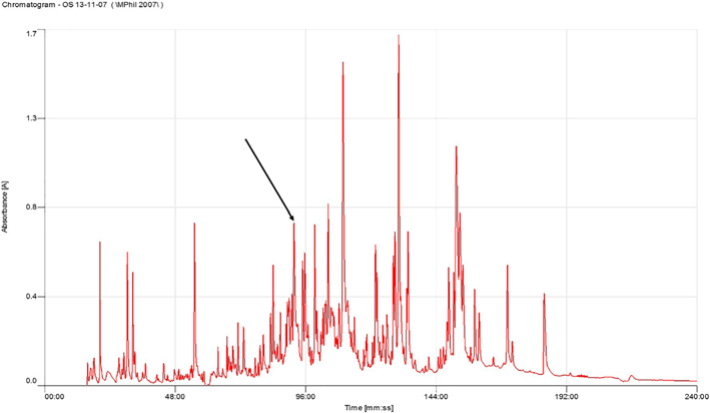
rpHPLC spectrum of crude *Odorrana.schmackeri* skin secretion Region of rpHPLC chromatogram of *O. schmackeri* skin secretion with arrow indicating the retention times (at 90 min) of the novel peptide OSTI. The detection wavelength was 214 nm with a flow rate of 1 ml/min in 240 min.

**Figure 2:**
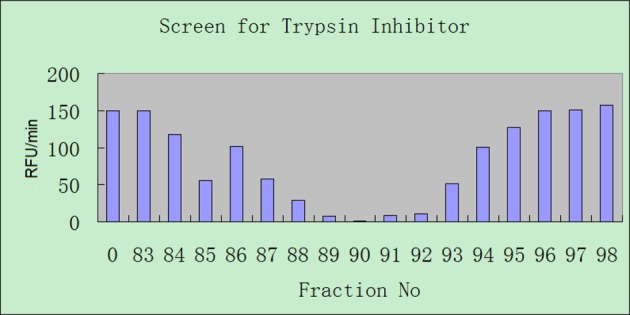
Trypsin inhibitory activity of rpHPLC fractions from *Odorrana.schmackeri* RpHPLC fractions that exhibited trypsin inhibitory activity. Sample 0 was the control with only added trypsin and substrate and did not include a fraction sample.

Skin secretions from the piebald odorous frog, *O. schmackeri*, were fractionated by rpHPLC using a C5 column (Figure 1). Samples of all 240 chromatographic fractions were subjected to a trypsin inhibition assay screen. Although inhibitory activity was detected in fractions 86–94, maximum activity was present in fraction 90 (Figure 2).

### Structural characterization and analysis of the novel peptide OSTI

**Figure 3:**
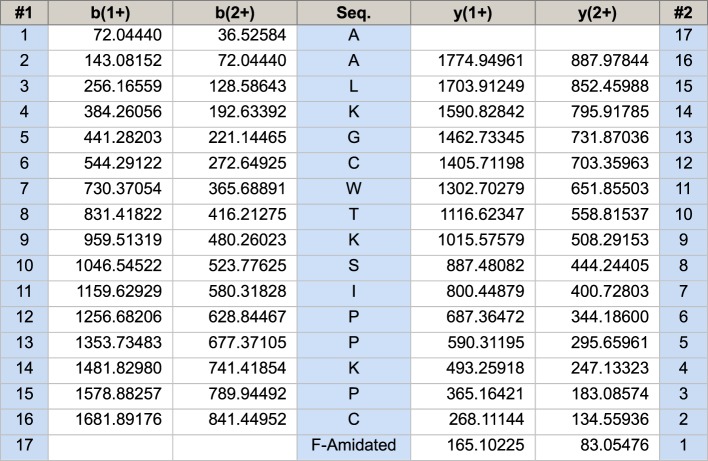
Predicted OSTI sequence from select rpHPLC fraction using LCQ-Fleet Expected single- and double- charged *b*- and *y*-ion series predicted from MS/MS fragmentation of OSTI. The fragment ions observed following actual fragmentation are shown in red (*b*-ions) and blue (*y*-ions) typefaces.

A single peptide with a single protonated molecular mass of 1803.41 Da was identified in chromatographic fraction 90 by using MALDI-TOF MS and this peptide was named OSTI. The LCQ-Fleet mass spectrometer in MS/MS mode, established the sequence of OSTI as: A-A-L/I-K-G-C-W-T-K-S-I/L-P-P-K-P-C-F-amide (Figure 3).

### Molecular cloning of OSTI cDNA and sequence analysis

**Figure 4:**
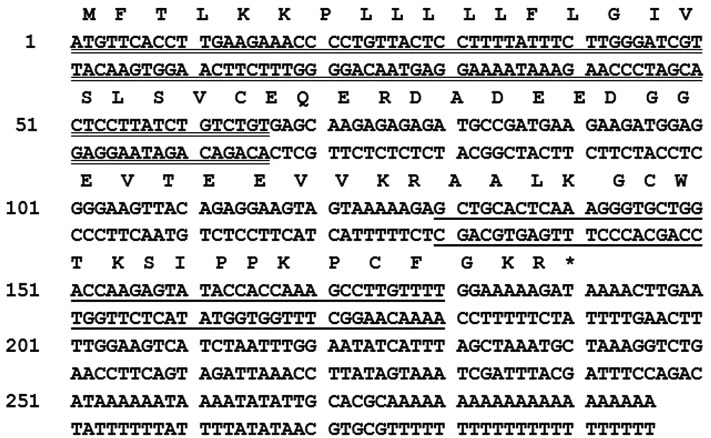
Translated Open Reading Frame (ORF) of OSTI from skin secretion of *Odorrana.schmackeri* Nucleotide and translated ORF amino acid sequences of cloned cDNA encoding the biosynthetic precursor of the trypsin inhibitory peptide, OSTI, from the skin secretion of the frog, *O. schmackeri*. The putative signal peptide is double-underlined, the mature active peptide is single-underlined and the stop codon is indicated by an asterisk.

From the skin-derived cDNA library, the cDNA encoding the biosynthetic precursor of OSTI was consistently and repeatedly cloned. The ORF of the cDNA consisted of 67 amino acid residues and the full primary structure of OSTI was confirmed and was present as a single copy located towards the C-terminus of the precursor (Figure 4). The sequence was preceded by two consecutive basic amino acids, Lys-Arg (KR), representing a typical processing site for endoproteolytic cleavage and immediately followed by a glycyl (G) residue amide donor and a second C-terminally located Lys-Arg processing site. The nucleotide sequence of the precursor-encoding cDNA of OSTI has been deposited in the EMBL Nucleotide Sequence Database under the accession code: LT615077.

### Peptide synthesis and MALDI-TOF MS confirmation of purity of synthetic OSTI and its analogue [Phe^9^]-OSTI

When the primary structure of OSTI had been unequivocally established, the peptide and its Phe^9^-substituted analogue were synthesized by standard solid-phase Fmoc chemistry using a Protein Technologies (Tucson, AZ, U.S.A.) PS3™ automated peptide synthesizer. Following cleavage from the synthesis resin, impurities were removed from the synthetic replicates by rpHPLC and the molecular masses of the purified major products were confirmed by MALDI-TOF MS (Supplementary Figures S1 and S2).

### Effects of synthetic OSTI on trypsin inhibition

**Figure 5:**
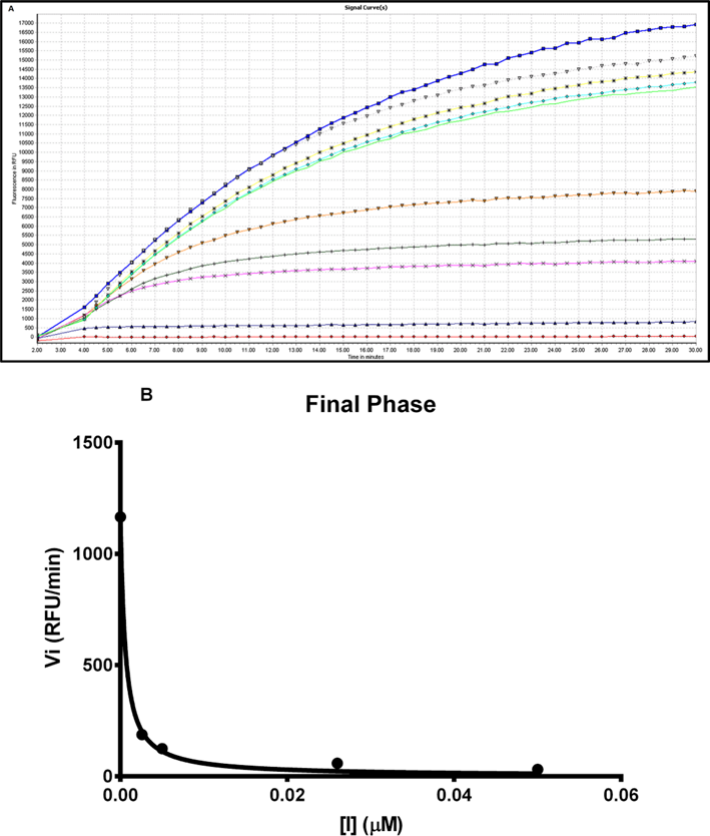
Trypsin inhibitory activity of OSTI (**A**) Progress curves for trypsin proteolysis in the presence of different concentrations of wild-type OSTI, 2.56 μM (
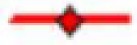
), 0.5 μM (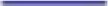), 0.095 μM (
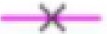
), 0.05 μM (
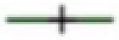
), 0.026 μM (
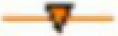
), 0.0073 μM (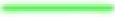), 0.005 μM (
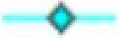
), 0.0026 μM (
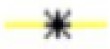
), 0.0095 μM (
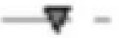
) and control (
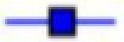
). (**B**) Final steady state rates (*V*_i_) for the trypsin-catalysed hydrolysis of Phe-Pro-Arg-NHMec (fixed concentration of 50 μM) in the presence of varying concentrations of OSTI (0–0.05 μM).

The synthetic replicate of OSTI was tested quantifiably for inhibitory activity against trypsin. The ‘progress curves’ for the hydrolysis of the fluorogenic substrate in the presence of competing concentrations of OSTI are shown in Figure 5A. These progress curves are typical of an inhibitor exhibiting reversible, slow-binding kinetics, where formation of product P_1_ (in this instance, NH_2_Mec) with time (t) is described by eqn (4).

(4) $$[{\text{P}}_{1}] =\frac{{\text{V}}_{\text{s}}\;\times \text{t}-({\text{V}}_{\text{s}}\;-{\text{V}}_{\text{o}}) \times (1-exp( -{\text{k}}_{\text{obs}}\cdot \text{t}))}{{\text{k}}_{\text{obs}}\;+\text{d}}$$

In this treatment, *V*_s_ is the final steady-state velocity, *V*_o_ is the initial velocity and *k*_obs_ is the apparent first order rate constant for the transition from initial to final steady state and d is a displacement term that reflects the concentration of product formed at *t*_0_. These progress curves indicate that the interaction of OSTI with trypsin follows a two-step complexing mechanism [19,20] as indicated below.


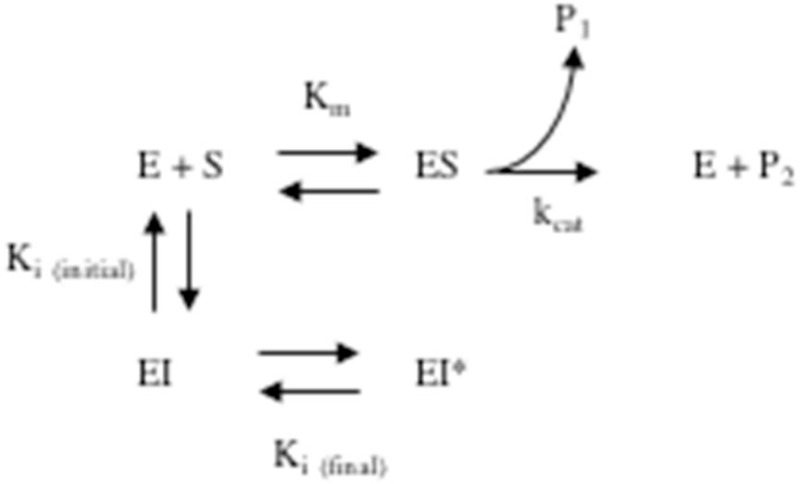


Values for the final steady-state velocity (*V*_s_) for product formation (P_1_) were estimated from these progress curves and were used to generate ‘Morison plots’ from which a *K*_i_ value of 0.3 ± 0.04 nM was determined for the inhibition of trypsin (Figure 5B).

### Effects of synthetic [Phe^9^]-OSTI on chymotrypsin inhibition

**Figure 6:**
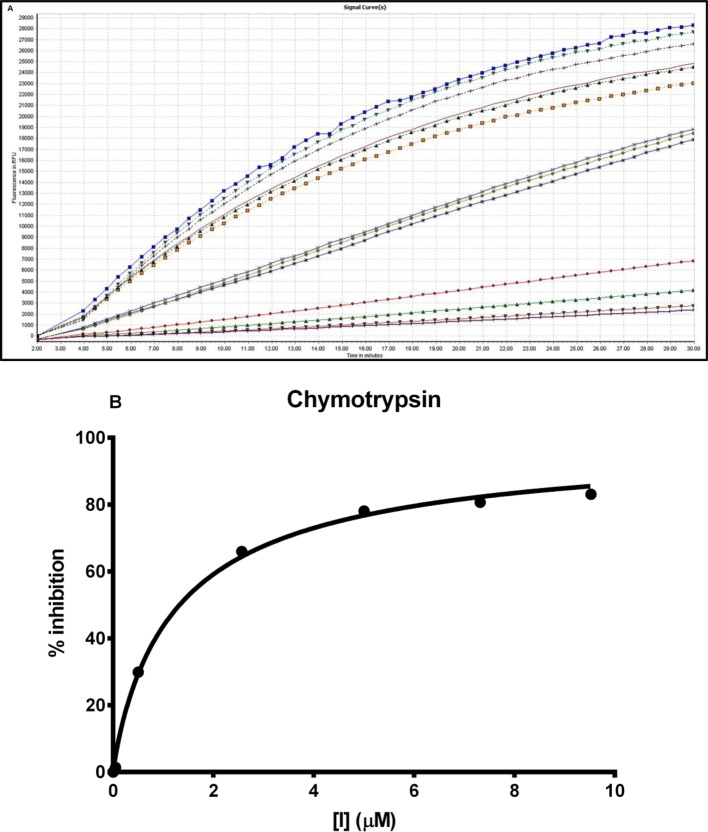
Chymotrypsin inhibitory activity of [Phe^9^]-OSTI (**A**) Progress curves for chymotrypsin proteolysis in the presence of different concentrations of [Phe^9^]-OSTI, 9.5 μM (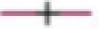), 7.3 μM (
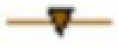
), 5 μM (
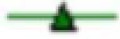
), 2.56 μM (
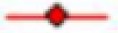
), 0.73 μM (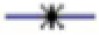), 0.95 μM (
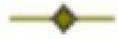
), 0.5 μM (
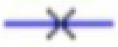
), 0.05 μM (
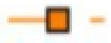
), 0.073 μM (
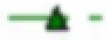
), 0.026 μM (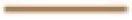), 0.005 μM (
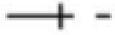
), 0.00256 μM (
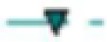
) and control (
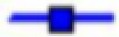
). (**B**) Plot of degree of inhibition (% relative to no peptide control) of chymotrypsin against concentration of [Phe^9^]-OSTI (0–9.5 µM). Data points were fitted to the curve, by non-linear regression analysis, using GraphPad Prism.

The synthetic replicate of [Phe^9^]-OSTI was tested quantifiably for inhibitory activity against chymotrypsin. The peptide was found to behave as a moderate inhibitor of chymotrypsin and ‘progress curves’ for the hydrolysis of the fluorogenic substrate Succinyl–Ala–Ala–Pro–Phe–NHMec in the presence of competing concentrations of peptide are shown in Figure 6A. Initial rates (*V*_i_) for product formation in the presence of [Phe^9^]-OSTI were estimated from these progress curves and these were compared with the initial rate (*V*_o_) of product formation in the absence of [Phe^9^]-OSTI. From these, the % inhibition obtained at each concentration of [Phe^9^]-OSTI studied was calculated and the data plotted (Figure 6B) in order to determine the IC_50_ value of the peptide for chymotrypsin. This [Phe^9^]-OSTI was then utilized, along with the determined *K*_m_ value for the chymotrypsin-catalysed hydrolysis of Succinyl–Ala–Ala–Pro–Phe–NHMec (used at a fixed concentration of 50 μM) to calculate a *K*_i_ value for the peptide, using eqn (3). This yielded a *K*_i_ of 1.0 ± 0.1 μM for the inhibition of chymotrypsin.

### Effects of synthetic OSTI on tryptase inhibition

**Figure 7:**
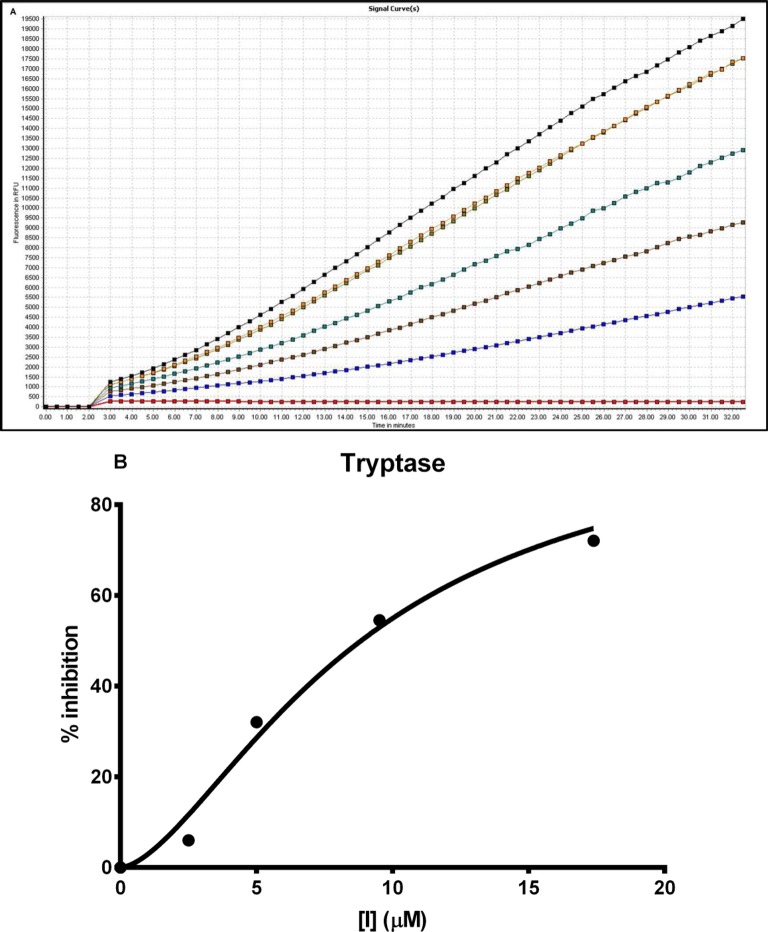
Tryptase inhibitory activity of OSTI (**A**) Progress curves for tryptase activity in the absence (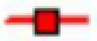) and presence of different concentrations of wild-type OSTI, 17.39 μM (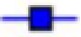), 9.52 μM (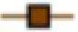), 5 μM (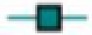), 2.56 μM (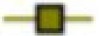) and control (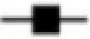). (**B**) Plot of degree of inhibition (% relative to no peptide control) of tryptase against concentration of OSTI (0–17.4 μM). Data points were fitted to the curve, by non-linear regression analysis, using GraphPad Prism.

The synthetic replicate of OSTI was also tested for inhibitory activity against tryptase. The peptide was found to behave as a moderate inhibitor of tryptase and ‘progress curves’ for the hydrolysis of the fluorogenic substrate Boc-Phe-Ser-Arg-NHMec in the presence of competing concentrations of peptide are shown in Figure 7A. Initial rates (*V*_i_) for product formation in the presence of OSTI were estimated from these progress curves and these were utilized as detailed for the inhibition of tryptase by OSTI to generate a IC_50_ plot (see Figure 7B), from which a *K*_i_ value of 2.5 ± 0.6 μM was obtained for the inhibition of tryptase by this synthetic replicate of OSTI.

A comparison of *K*_i_ values for trypsin, chymotrypsin and tryptase obtained for wild-type OSTI and its analogue [Phe^9^]-OSTI, is shown in Table 1. The site of lysine substitution in the analogue is underlined. Data represent the mean ± S.E.M. of three independent experiments, each performed in duplicate.

**Table 1:** A comparison of OSTI and [Phe^9^]-OSTI against trypsin, chymotrypsin and tryptase

Peptide name	Sequence	Net charge	Trypsin inhibition *K*_i_	Chymotrypsin inhibition *K*_i_	Tryptase inhibition *K*_i_
OSTI	AALKGCWT**K**SIPPKPCF-amide	3.9	0.3 ± 0.04 nM	N/A	2.5 ± 0.6 μM
[Phe9]-OSTI	AALKGCWT**F**SIPPKPCF-amide	2.9	N/A	1.0 ± 0.1 μM	N/A

The substitutions of Lys^9^ with a phenylalanine residue decreased the net charge by +1. The result was the almost total loss of trypsin inhibition activity.

### Secondary structures and physicochemical properties of OSTI and its analogue

**Figure 8:**
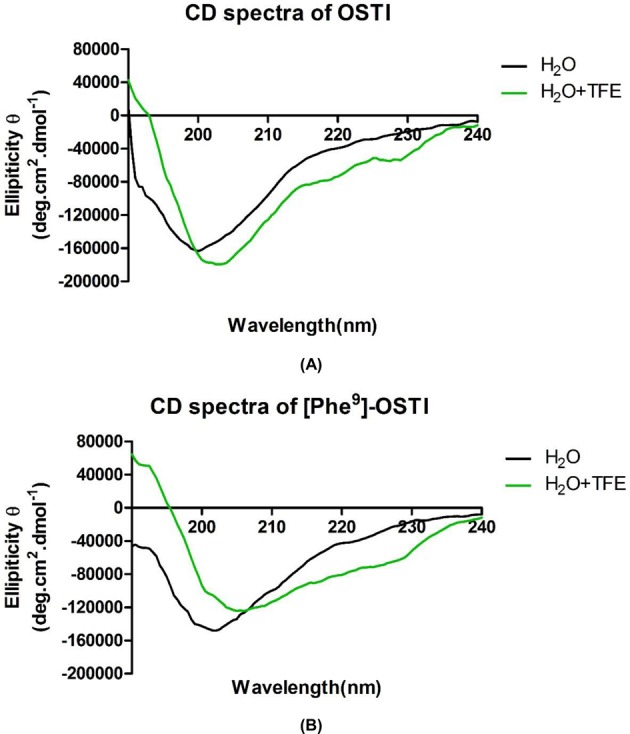
Circular dichroism spectra of OSTI and [Phe^9^]-OSTI The CD spectra recorded for OSTI and [Phe^9^]-OSTI (100 μM) in (**A**) 10 mM ammonium acetate water solution and (**B**) 50% 2,2,2-TFE/10 mM ammonium acetate water solution.

The CD spectra of OSTI and [Phe^9^]-OSTI indicate that their structures in aqueous situation are similar, while in TFE-water solutions are slightly different (Figure 8). In aqueous solution, both OSTI and [Phe^9^]-OSTI displayed a mixed conformation of 51% β-sheet and 42% random coil based on the CD spectra. In contrast, in 50% TFE-water solution, the main structure of these two peptides changed from β-sheet to random coil. Specifically, OSTI adopted a mixture conformation of α-helix (8%), β-sheet (45%) and random coil (47%), while [Phe^9^]-OSTI presented a mixture conformation of α-helix (9%), β-sheet (36%) and random coil (55%).

## Discussion

The BBIs are abundant in many plants, such as soybeans, many other leguminous plants and sunflowers. Amphibian skin secretions have proven to be a rich source of biologically active peptides and proteins, including BBIs, such as HV-BBI from the Chinese Bamboo odorous frog, *Huia versabilis* [9] and HJTI from *O. hejiangensis* [11].

In this project, a novel BBI named OSTI, with the primary structure, AALKGCWTKSIPPKPCF-amide, was isolated and characterized from the skin secretion of the piebald odorous frog, *O. schmackeri*, and it contains a canonical Bowman–Birk-type protease inhibitor loop between Cys^6^ and Cys^16^. Synthetic wild-type OSTI and its residue-9 phenylalanine-substituted analogue, were subjected to a series of pharmacological assays and their differences and potential applications are discussed here.

Many publications have reported that the core disulfide constrained reactive site loop of BBI and some residues in this loop, including lysine at P_1_ position, threonine at P_2_ position, serine at P_1_′ position and proline at P_3_′ position, are highly conserved. As the sequence of the reactive site determines the specificity of the inhibition, based on the elegant research of many scientists, the P_1_ position lysine residue is optimal for trypsin inhibition and the phenylalanine is optimal for chymotrypsin inhibition [7]. The results of protease inhibition assays using OSTI and [Phe^9^]-OSTI, confirmed this differentiation. The mechanism of action of protease inhibitors is achieved through cognate enzyme binding in a substrate-like manner and the specificity is determined by whether the P_1_ position residue can fit into the S1 pocket of proteases. In the S1 pocket of trypsin, there is a negatively charged aspartic acid residue that prefers long and positively charged side-chain residues and in the S1 pocket of chymotrypsin, there is a serine residue that prefers a deep hydrophobic pocket, so long uncharged side-chained amino acids like phenylalanine, are optimal. Besides simple amino acid effects, the precise mechanisms of inhibitor interactions with proteases must be associated with their steric conformation, which is why not all the arginine and lysine residues containing sequences have the ability to inhibit trypsin and phenylalnine containing peptides to inhibit chymotrypsin. Their secondary structure, including the conserved disulfide bridge, is essential for bioactivity. There are reports that the core disulfide loop of the vast majority of BBIs forms a short type VIb β-turn motif and it is extremely well ordered for a short peptide. While our CD data revealed that OSTI and [Phe9]-OSTI did not display canonical β-turn motif in aqueous solution and only OSTI formed low percentage of β-sheet secondary structure in 50% TFE-water solution, this might be the consequence of a solution-dependent inherent property of the peptides. In addition, OSTI is highly analogous to another peptide HV-BBI, which we have previously demonstrated to be an excellent trypsin inhibitor [9]. Recent X-ray crystallographic studies have demonstrated that HV-BBI binds to the active site of trypsin, with the -Thr-Lys-Ser- triplet of the canonical loop (present in OSTI and HV-BBI) occupying the S2-S1-S1′-binding pockets of trypsin [21], therefore, to suggest that OSTI behaves as a competitive reversible inhibitor. While the conformation of OSTI and [Phe^9^]-OSTI remain to be further investigated by NMR under more complex environments, this could shed some light on exactly how they inhibit proteases in a competitive and/or non-competitive manner [22].

Tryptase and chymase are atypical serine proteases that are synthesized, stored and released by mast cells. Due to their close involvement in the pathogenesis of inflammation and some other diseases, there have been reports of BBIs possibly having utility in the treatment of such conditions. In the present study, we tested the activity of OSTI in a tryptase inhibition assay and found a relatively fast association rate constant of 8.36 μM. These data were not in agreement with the conclusion put forward by Ware et al. [23], whose study focused on the interaction between tryptase, chymase and soybean Bowman–Birk protease inhibitors. They believed that human tryptase was not effectively inhibited by protein protease inhibitors, while in our study, it was obvious that OSTI could achieve this very effectively. It could be that the active site of tryptase might be restricted in a certain way which makes it difficult for large protein inhibitors to access, but suitable for much smaller sized BBI peptides. Human mast cell chymase is another protease that could be inhibited by BBIs based on previous studies [23]. As the BBI is a two-headed protease inhibitor and the work of Ware et al. [23] reported a 1:1 stoichiometry of inhibition, this probably implies that only one domain of the two-headed reactive sites takes part in the inhibition activity and the chymotrypsin inhibitory domain presents the highest possibility. Thus, the [Phe^9^]-OSTI analogue that showed effective chymotrypsin inhibition ability would probably inhibit chymase as well. Despite that, other factors should be taken into consideration, for instance the kinetic data based on inhibition of chymase by soybean BBI have identified a non-competitive mechanism through a Lineweaver–Burk double-reciprocal plot [24]. Also, the preferred cleavage substrate of chymase contains a large aliphatic amino acid residue in the P_1_ position that seems to be optimal for chymase interactions [25,26]. As has been mentioned above, this is consistent with the leucine residue’s ranking being in third place for affinity at the P_1_ position for chymotrypsin inhibition activity. In this respect, [Phe^9^]-OSTI does not meet the criteria to be a chymase inhibitory peptide and a [Leu^9^]-OSTI might have been a better choice. All these results and predictions might be useful for development of therapeutics for the treatment of allergic inflammatory reactions.

Skin secretion BBIs are believed to be widespread in species of frogs of the *Rana* (*Odorrana*) genus and their primary structure-based modifications can produce a series of analogues that exhibit substantial bioactivities, such as canonical serine protease inhibition, myotropic activity, anticarcinogenic activity or even antimicrobial activity. Perhaps the most interesting discovery in this project is the strong tryptase inhibition activity of OSTI, as numerous biological and immunological investigations have implicated tryptase as a regulator in the pathology of a variety of allergic and inflammatory conditions including rhinitis, conjunctivitis and most notably asthma, and thus, identification of a potent and selective tryptase inhibitor could provide avenues for novel drug development. Future systematic structure/activity studies may represent a way forward for generation of peptides with desirable therapeutic endpoints for a variety of diseases.

## Compliance with ethical standards

This research did not involve any human participants.

## Abbreviations

BBI: Bowman–Birk-type inhibitor
MALDI-TOF: matrix-assisted laser desorption ionization time-of-flight
NUP: nested universal primer
OSTI: *Odorrana schmackeri* Trypsin Inhibitor
pLR: peptide leucine arginine
RACE: rapid amplification of cDNA ends
rpHPLC: reverse phase HPLC
TFA: Trifluoroacetic acid
TFE: trifluoroethanol

## References

[1] Chen T.B. and Shaw C. (2003) Identification and molecular cloning of novel trypsin inhibitor analogs from the dermal venom of the Oriental fire-bellied toad (*Bombina* * orientalis*) and the European yellow-bellied toad (*Bombina variegata*). Peptides 24, 873–880 12948839 10.1016/s0196-9781(03)00165-7

[2] Birk Y. (1985) The Bowman-Birk inhibitor. Trypsin- and chymotrypsin-inhibitor from soybeans. Int. J. Pept. Protein Res. 25, 113–131 3886572 10.1111/j.1399-3011.1985.tb02155.x

[3] Prakash B., Selvaraj S., Murthy M.R.N., Sreerama Y.N., Rao D.R. and Gowda L.R. (1996) Analysis of the amino acid sequences of plant Bowman-Birk inhibitors. J. Mol. Evol. 42, 560–569 8662008 10.1007/BF02352286

[4] Song H.K., Kim Y.S., Yang J.K., Moon J., Lee J.Y. and Suh S.W. (1999) Crystal structure of a 16 kDa double-headed Bowman-Birk trypsin inhibitor from barley seeds at 1.9 angstrom resolution. J. Mol. Biol. 293, 1133–1144 10547291 10.1006/jmbi.1999.3239

[5] Nishino N., Aoyagi H., Kato T. and Izumiya N. (1975) Synthesis and activity of nonapeptide fragments of soybean Bowman-Birk inhibitor. Experientia 31, 410–412 1168143 10.1007/BF02026346

[6] Laskowski M. Jr and Kato I. (1980) Protein inhibitors of proteinases. Annu. Rev. Biochem. 49, 593–626 6996568 10.1146/annurev.bi.49.070180.003113

[7] Odani S. and Ono T. (1980) Chemical substitutions of the reactive site leucine residue in soybean Bowman-Birk proteinase-inhibitor with other amino-acids. J. Biochem. 88, 1555–1558 7462194 10.1093/oxfordjournals.jbchem.a133126

[8] McBride J.D., Brauer A.B.E., Nievo M. and Leatherbarrow R.J. (1998) The role of threonine in the P-2 position of Bowman-Birk proteinase inhibitors: studies on P-2 variation in cyclic peptides encompassing the reactive site loop. J. Mol. Biol. 282, 447–458 9735299 10.1006/jmbi.1998.2032

[9] Song G.H., Zhou M., Chen W., Chen T.B., Walker B. and Shaw C. (2008) HV-BBI-A novel amphibian skin Bowman-Birk-like trypsin inhibitor. Biochem. Biophys. Res. Commun. 372, 191–196 18486596 10.1016/j.bbrc.2008.05.035

[10] Lin Y., Hu N., Lyu P., Ma J., Wang L., Zhou M. (2014) Hylaranins: prototypes of a new class of amphibian antimicrobial peptide from the skin secretion of the oriental broad-folded frog, *Hylarana latouchii*. Amino Acids 46, 901–909 24378871 10.1007/s00726-013-1655-1

[11] Wang H., Wang L., Zhou M., Yang M., Ma C.B., Chen T.B. (2012) Functional peptidomics of amphibian skin secretion: a novel Kunitz-type chymotrypsin inhibitor from the African hyperoliid frog, Kassina senegalensis. Biochimie 94, 891–899 22197669 10.1016/j.biochi.2011.12.008

[12] Tyler M.J., Stone D.J.M. and Bowie J.H. (1992) A novel method for the release and collection of dermal, glandular secretions from the skin of frogs. J. Pharmacol. Toxicol. 28, 199–200 10.1016/1056-8719(92)90004-k 1296824

[13] Di Tommaso S., Rotureau P., Crescenzi O. and Adamo C. (2011) Oxidation mechanism of diethyl ether: a complex process for a simple molecule. Phys. Chem. Chem. Phys. 13, 14636–14645 21735019 10.1039/c1cp21357a

[14] Morrison J.F. (1969) Kinetics of the reversible inhibition of enzyme-catalysed reactions by tight-binding inhibitors. Biochim. Biophys. Acta 185, 269–286 4980133 10.1016/0005-2744(69)90420-3

[15] Cheng Y. and Prusoff W.H. (1973) Relationship between the inhibition constant (K1) and the concentration of inhibitor which causes 50 per cent inhibition (I50) of an enzymatic reaction. Biochem. Pharmacol. 22, 3099–3108 4202581 10.1016/0006-2952(73)90196-2

[16] Lobley A., Whitmore L. and Wallace B.A. (2002) DICHROWEB: an interactive website for the analysis of protein secondary structure from circular dichroism spectra. Bioinformatics 18, 211–212 11836237 10.1093/bioinformatics/18.1.211

[17] Whitmore L. and Wallace B.A. (2004) DICHROWEB, an online server for protein secondary structure analyses from circular dichroism spectroscopic data. Nucleic Acids Res. 32, W668–W673 15215473 10.1093/nar/gkh371 PMC441509

[18] Whitmore L. and Wallace B.A. (2008) Protein secondary structure analyses from circular dichroism spectroscopy: methods and reference databases. Biopolymers 89, 392–400 17896349 10.1002/bip.20853

[19] Cha S. (1975) Tight-binding inhibitors-I. Kinetic behavior. Biochem. Pharmacol. 24, 2177–2185 1212266 10.1016/0006-2952(75)90050-7

[20] Morrison J.F. (1982) The slow-binding and slow, tight-binding inhibition of enzyme-catalyzed reactions. Trends Biochem. Sci. 7, 102–105

[21] Grudnik P., Debowski D., Legowska A., Malicki S., Golik P., Karna N. (2015) Atomic resolution crystal structure of HV-BBI protease inhibitor from amphibian skin in complex with bovine trypsin. Proteins 83, 582–589 25546528 10.1002/prot.24750

[22] Brauer A.B.E., Kelly G., McBride J.D., Cooke R.M., Matthews S.J. and Leatherbarrow R.J. (2001) The Bowman-Birk inhibitor reactive site loop sequence represents an independent structural beta-hairpin motif. J. Mol. Biol. 306, 799–807 11243789 10.1006/jmbi.2000.4410

[23] Ware J.H., Wan X.S., Rubin H., Schechter N.M. and Kennedy A.R. (1997) Soybean Bowman-Birk protease inhibitor is a highly effective inhibitor of human mast cell chymase. Arch. Biochem. Biophys. 344, 133–138 9244390 10.1006/abbi.1997.0182

[24] Fukusen N., Kato Y., Kido H. and Katunuma N. (1987) Kinetic-studies on the inhibitions of mast-cell chymase by natural serine protease inhibitors: indications for potential biological functions of these inhibitors. Biochem. Med. Metab. Biol. 38, 165–169 3479119 10.1016/0885-4505(87)90076-4

[25] Kinoshita A., Urata H., Bumpus F.M. and Husain A. (1991) Multiple determinants for the high substrate-specificity of an angiotensin II forming chymase from the human heart. J. Biol. Chem. 266, 19192–19197 1918036

[26] Powers J.C., Tanaka T., Harper J.W., Minematsu Y., Barker L., Lincoln D. (1985) Mammalian chymotrypsin-like enzymes. Comparative reactivities of rat mast-cell proteases, human and dog skin chymases, and human cathepsin-g with peptide 4-nitroanilide substrates and with peptide chloromethyl ketone and sulfonyl fluoride inhibitors. Biochemistry 24, 2048–2058 3893542 10.1021/bi00329a037

